# Family Functioning in Families of Adolescents with Mental Health Disorders: The Role of Parenting Alliance

**DOI:** 10.3390/children8030222

**Published:** 2021-03-13

**Authors:** Sofía Baena, Lucía Jiménez, Bárbara Lorence, Mᵃ Victoria Hidalgo

**Affiliations:** Department of Developmental and Educational Psychology, Faculty of Psychology, University of Seville, 41018 Seville, Spain; luciajimenez@us.es (L.J.); bll@us.es (B.L.); victoria@us.es (M.V.H.)

**Keywords:** adolescence, coparenting, family processes, mental health disorders, public health

## Abstract

This study primarily examined the predictive role of emotional and behavioral disorders in family cohesion and the moderating role of parenting alliance. Adolescents’ mental health issues are a major concern, with important implications for individuals and their families. However, the impact of mental disorders on family processes has been less widely studied. Participants in this study were 72 parents of adolescent beneficiaries of mental health services. Questionnaires assessed family cohesion, parenting alliance, and sociodemographic factors. Results indicated that emotional and behavioral disorders did not have an influence on family cohesion. They also suggested that parenting alliance may be a protective factor for family cohesion. This paper highlights the role of parenting alliance as a potential protective factor in positive family processes. These findings support the importance of focusing on the parental subsystem in therapy, and the need to incorporate a positive parenting perspective when working with these families.

## 1. Introduction

Mental disorders during adolescence are a major health concern. The latest epidemiological studies consider mental disorders to be the most common cause of non-fatal illness, with a particularly high prevalence during adolescence [1]. The presence of a mental disorder during this stage can impact adolescents’ socialization processes [2] and is considered a potential precursor of adult psychopathology [2,3].

Adolescence is a particularly vulnerable developmental period [4]. At an individual level, adolescence involves biological, psychological, and social changes, together with increased rebellious conducts, resistance to parental authority, and emotional instability [5]. At a family level, adolescence is an important transition period in which parents face new challenges. The family system has the difficult task of balancing the preservation of family unity with adolescents’ need for autonomy and differentiation [6]. Adolescence is therefore a particularly stressful transition for families, in which the number of conflicts tends to increase [4,7]. These challenges are even more evident in the families of adolescents with mental disorders, who face both difficulties associated with the transition and those connected to the mental health problem.

The transactional and systemic approach views the family context as being characterized by reciprocal interactions [8]. General systems theory states that every change in one member of the system will in turn influence every other member, with the relationship between parents’ behavior and children’s internalizing and externalizing problems being bidirectional and reciprocal [9]. However, despite these theoretical assumptions, classical studies have focused solely on parent-to-child effects, with the literature consistently demonstrating the influence that parent and family dimensions have on child psychopathology [10,11].

Despite the contributions made by the parent-to-child perspective, there is currently increased interest in child-to-parent and bidirectional effects. Within this perspective, research has focused on the relationship between externalizing problems and parenting practices during childhood and adolescence. The results are somewhat ambiguous, with some authors finding bidirectional relationships [9,12], others parent-to-child effects [11,13], and others child-to-parent effects [10,14].

Although family dysfunction is one of the main predictors of psychopathology [13,15], detailed studies of the influence of mental disorders on family functioning during adolescence are scarce and focus mainly on family conflict [5,16]. Considering the increasingly central role played by adolescents in family processes, it is essential to expand our understanding of the child-to-parent perspective in this developmental stage. Lubenko and Sebre [5] claim that adolescents’ behavioral problems influence family dynamics, increasing family conflict and decreasing family cohesion. Brière, Archambault, and Janosz [16] have found reciprocal associations between adolescents’ depressive symptoms and family conflict, but only child-to-parent effects on communication. These studies highlight the importance of continuing to explore how the families of adolescents with mental disorders function, since different mental disorders may influence family dynamics in different ways. It is also important for analyses to incorporate specific aspects of family functioning, rather than a general assessment, and to examine the impact of mental disorders on positive facets of family life, such as family cohesion. This process, defined as emotional closeness between family members [17], is an important positive element of family functioning [18] as well as a relevant protective factor for families with mental health disorders [19,20]. However, few studies have addressed the impact of mental health disorders on this dimension.

Parenting alliance or coparenting is defined as the united action of parents in their parental role [21] and is considered a central element of family life [22,23]. Adolescence is a particularly difficult period that involves new challenges in the coparenting relationship [24]; however, little is known about coparenting processes during this period.

From the parent-to-child perspective, evidence supports the impact of coparenting on children’s adjustment [24,25] as a buffer against the development of externalizing [24,26] and internalizing problems [24,27]. Parenting alliance also plays a mediating role in family processes [23]. However, further research into this dyadic dimension is required and there is as yet little evidence of its potential buffering effect from the child-to-parent perspective. Cui, Donnellan, and Conger [28] have argued that adolescents’ behaviors may impact family functioning through their influence on the coparenting relationship. Other authors have highlighted the influence of emotional and behavioral disorders on the coparenting relationship [29,30]. Results indicate that parents of children and adolescents with behavioral or emotional problems either have higher levels of conflict in their coparenting relationship [24,29,30], or come together, engaging in more supportive behaviors [28].

The purpose of this paper is to further our understanding of the influence of adolescents’ mental disorders on family dynamics. Specifically, the aims of the present study are:

1. To analyze the predictive role of emotional/behavioral disorders and parenting alliance on family cohesion.
2. To examine the moderating effect of parenting alliance on the relationship between emotional and behavioral disorders and family cohesion.

## 2. Materials and Methods

### 2.1. Participants

Participants were 72 parents of adolescent beneficiaries of specialized mental health services in Spain. The adolescents were evenly distributed in terms of gender (56.60% males), with ages ranging between 12 and 17 (mean age (Mage) = 14.38, standard deviation (SD) = 1.36). Parents (53.98% mothers, 40.71% fathers, 5.31% stepparents) ranged from 25 to 58 years old, with a mean age of 44.43 (SD = 5.12). In the vast majority (94.30%) of families, both parents lived together, while in 5.70%, they either lived separately or were absent (deceased or no relationship with their children).

### 2.2. Measures

Firstly, the family cohesion subscale (10 items) of the Family Cohesion and Adaptability Scales [31] was used to evaluate family cohesion, defined as the emotional bond among family members. This scale ranges from 1 (never or almost never) to 5 (almost always), with higher scores indicating more family cohesion. Cronbach’s alpha for this study was 0.77. Secondly, a Spanish version of the 20-item Parental Alliance Inventory [32] was used to evaluate parenting alliance, defined as a couple’s supportive relationship in their parental role. In the present study, we used the global score, with higher scores indicating greater parenting alliance. All items were scored using a 5-point Likert-type scale, with values ranging from 1 (strongly disagree) to 5 (strongly agree). Cronbach’s alpha for this study was 0.93. Finally, we also used an ad hoc sociodemographic questionnaire designed to record adolescents’ and parents’ gender and age. Mental health providers completed clinical diagnoses of the adolescents.

### 2.3. Procedure

All families referred to a family therapy intervention in a specialized mental health service in the south of Spain were deemed eligible to participate in the study. The inclusion criteria for the program were: (1) one child from the family had to be a beneficiary of the mental health service, (2) the mental health provider had to have detected a need for therapeutic intervention with the child’s family, based on clinical judgment, and (3) the child had to have been diagnosed with a mental disorder according to the ICD-10 (International Statistical Classification of Diseases and Related Health Problems). Participants had to meet all three criteria to be included in the program. Following referral to the program, the mental health provider contacted the family to inform them of the study. Once the families had agreed to participate, we contacted them directly to set up an appointment. Most referred families (61.40%) participated in the study. The main reason for non-participation was our inability to contact the family.

Parents completed a battery of questionnaires about family dynamics before the start of the intervention, and mental health providers sent details of the adolescents’ diagnoses. Ethical standards were followed at all times: participation was strictly voluntary, and the aims of the study were clearly explained, and participants were assured of the anonymity of their responses and signed an informed consent document. The study was approved by the research ethics committee at the local hospital (protocol 22/0509).

### 2.4. Data Analyses

The statistical package SPSS vs. 22 was used. We tested multiple linear hierarchical regression models to examine the role of adolescents’ disorders and parenting alliance on family cohesion, as well as to observe possible interaction effects. We included the variables in different steps, controlling for adolescents’ age and gender. When the interaction was found to be significant, we performed an analysis of the simple regression slopes.

Assumptions of linearity, normality, homoscedasticity, and non-multicollinearity were examined, with satisfactory results. We used the squared semi-partial correlation as an effect size index.

## 3. Results

With the aim of grouping the diagnoses into comparable categories, we established two restrictive groups that were consistent with the ICD-10 codes corresponding to the most significant types of disorder with onset during childhood or adolescence: Internalizing problems (referred to here as emotional disorders; *n* = 20) and externalizing problems (referred to here as behavioral disorders; *n* = 28). This grouping also allowed us to analyze how different symptomatic manifestations influence family dynamics, with one category representing more disruptive behaviors and other more internal manifestations. In the case of comorbidity, if the disorders in question belonged to different groups (e.g., comorbidity between conduct and anxiety disorders), the mental health provider decided which was the most salient at the time of the study.

### Predictive Role of Disorders on Family Cohesion

We tested two different regression models, including the independent variables in the regression equation in four successive steps: (1) child characteristics (age and gender) as control variables, (2) behavioral disorders/emotional disorders (0 = absence, 1 = presence), (3) parenting alliance, and (4) disorder X parenting alliance interaction.

The final model with behavioral disorders as predictors (see Table 1) explained 45.5% of the variance (*F*(5, 67) = 13, *p* < 0.001). The model indicated no significant direct effects of behavioral disorders, although it did indicate a significant direct effect of both child’s age and parenting alliance on family cohesion, with the latter explaining 32% of the variance. Child’s age was negatively associated with family cohesion, whereas parenting alliance was positively associated with this variable. Finally, the interaction effect between the disorder and parenting alliance significantly explained 5.5% of family cohesion.

**Figure 1:**
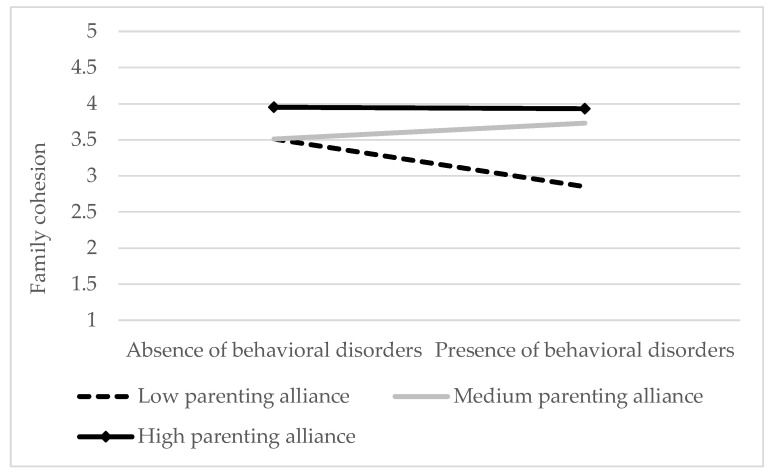
Interaction between behavioral disorders and parenting alliance for family cohesion.

As the interaction effect was significant, we analyzed the simple regression slopes. The moderating variable was divided into three groups based on standard deviation (±1 SD). According to the recommendations made by Aiken and West [33], the results indicated that the relationship between behavioral disorders and family cohesion was significant only for low (*t* = −2.36, *p* < 0.001) levels of parenting alliance, not for high (*t* = 1.94, *p* = 0.06) or medium levels (*t* = −0.22, *p* = 0.83). To present the results more clearly, we divided the moderating variable (parenting alliance) into three groups (taking as a reference the 33rd and the 66th percentiles) and used the two levels of the predictive variable (absence or presence of a behavioral disorder). Subsequently, we performed an analysis of variance (ANOVA), which revealed statistically significant differences in family cohesion in the presence and absence of a behavioral disorder when parenting alliance levels were low (*F*(1, 25) = −2.88, *p* < 0.001, *R*^2^ = 0.44), but not when they were either medium *(F*(1, 21) = 0.28, *p* = 0.245, *R*^2^ = 0.06) or high (*F*(1, 25) = <0.01, *p* = 0.948, *R*^2^ = < 0.01). As shown in Figure 1, when parenting alliance levels were high and medium, family cohesion levels were stable in both the presence and absence of a disorder, whereas a significant decrease in family cohesion was observed in the presence of a behavioral disorder when parenting alliance was low.

The final model with emotional disorders as a predictor (see Table 2) explained 44% of the variance (*F*(5, 67) = 12.30, *p* < 0.001). The results indicated no significant direct effects of emotional disorders, although they did indicate a significant effect of parenting alliance, which was the main predictor of family cohesion, explaining 36% of the variance. Parenting alliance was positively associated with family cohesion. Finally, the interaction effect between the disorder and parenting alliance significantly explained 4% of family cohesion.

**Figure 2:**
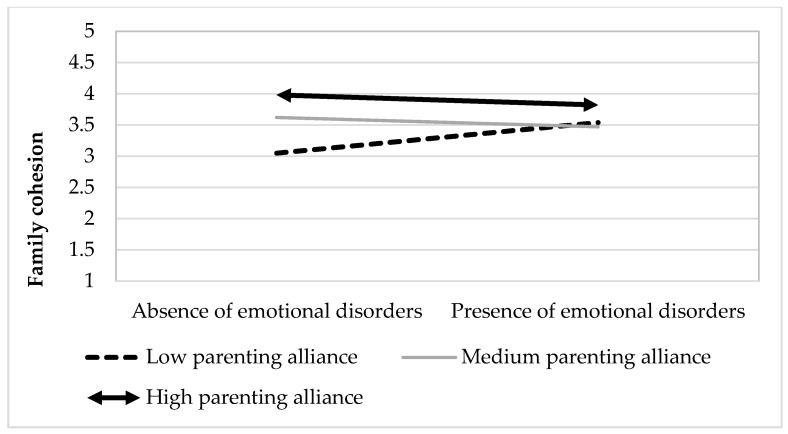
Interaction between emotional disorders and parenting alliance for family cohesion.

Since the interaction effect was also significant in this model, we analyzed the simple regression slopes. As in the previous model, the moderating variable was divided into three groups based on standard deviation (±1 SD). The results indicated that the relationship between emotional disorders and family cohesion was significant only for low (*t* = 2.37, *p* = 0.02) but not for medium (*t* = 0.57, *p* = 0.57) or high levels of parenting alliance (*t* = −1.59, *p* = 0.12). To present the results more clearly, we again divided the moderating variable (parenting alliance) into three groups (taking as a reference the 33rd and the 66th percentiles) and used the two levels of the predictive variable (absence or presence of an emotional disorder). Subsequently, we performed an ANOVA, which revealed significant differences in family cohesion in the presence and absence of an emotional disorder when parenting alliance levels were low (*F*(1, 25) = 7.19, *p* = 0.013, *R*^2^ = 0.22), but not when they were medium (*F*(1, 21) = 0.25, *p* = 0.620, *R*^2^ = 0.01) or high (*F*(1, 25) = 0.46, *p* = 0.505, *R*^2^ = 0.02). As shown in Figure 2, when parenting alliance was high and medium, family cohesion levels remained stable in both the presence and absence of a disorder, while when parenting alliance levels were low, a significant increase in family cohesion was observed in the presence of an emotional disorder.

## 4. Discussion

The aim of this study was to analyze the predictive role of emotional and behavioral disorders and parenting alliance on family cohesion, along with the possible moderator effect of parenting alliance. The results indicate that neither behavioral nor emotional disorders have a direct influence on positive aspects of family functioning, such as family cohesion. This finding is not consistent with the extant literature, which reports evidence of the impact of mental disorders on family functioning [5,16]. However, previous studies have focused on the influence of disorders on either global family functioning [34] or on family conflict [16], overlooking the positive elements of family life. Our results may be explained by the fact that our study design took the different aspects that define family functioning into consideration separately, rather than analyzing it as a unitary construct. It is possible that mental health disorders may have an impact on general or global family functioning, while not influencing specific elements of family life. Therefore, whereas behavioral or emotional disorders may increase family conflict [5,16] and communication difficulties [16], other (more positive) aspects, such as family unity, may remain stable. These results are consistent with previous research on normative parent–adolescent relationships, which has found that these relationships may remain close and affectionate despite increased conflict [18]. It is therefore important to continue exploring and analyzing the impact of mental disorders on specific aspects of family functioning, both positive and negative, so as to be able to draw more definite and accurate conclusions about how these disorders influence family life and design more targeted interventions.

Our results also indicate the importance of parenting alliance for family cohesion. This is consistent with that reported by McHale [35], who found the parental subsystem to be a transformer of family processes. A supportive relationship between parents is fundamental to family cohesiveness, with conflictive relationships being one of the main reasons for family cohesion dissolution [36]. Feinberg [37] considers coparenting to be a central element in family life, acting as a possible mediator and moderator to the influence of risk at the individual or family level, on family outcomes.

Finally, the interaction between behavioral and emotional disorders and parenting alliance was significant. In accordance with Luthar’s model [38], our results indicate that parenting alliance has a protective-stabilizing effect on family cohesion in the presence of a behavioral disorder. This finding is consistent with results reported within the parent-to-child perspective, in which parenting alliance has been found to have a buffering effect [24]. High and medium levels of parenting alliance also have a stabilizing effect in the presence of an emotional disorder, whereas low levels of parenting alliance are associated with an increase in family cohesion. These findings may be explained by general systems theory, which argues that systems tend towards homeostasis. According to the systemic family approach, the symptoms manifested by any member of the family may have an unconscious and non-intentional homeostatic effect on the entire family system [39]. Taking this approach as our reference, emotional disorders may help keep the family together when unity is at risk, thereby maintaining the balance. These results point towards similarities in how different disorders influence family dynamics, since, for example, neither emotional nor behavioral disorders had a direct influence on family cohesion. However, they also highlight certain differences in the way in which they influence family cohesion, particularly in relation to the role of parenting alliance.

## 5. Conclusions

In conclusion, the findings reported here indicate that emotional and behavioral disorders do not have a direct effect on positive aspects of family functioning, such as family cohesion, suggesting that despite difficulties, family unity is preserved. Moreover, they also provide evidence supporting the essential role played by parenting alliance in family cohesion, as well as its protective and stabilizing effect in relation to the impact of behavioral disorders on families.

## Figures and Tables

**Table 1:** Hierarchical regression model coefficients for behavioral disorders and parenting alliance predicting family cohesion.

Step	β	*t/F*	Δ*F*	*^a^sr*^2^/Δ*R*^2^
1		3.19	3.19	0.06
Child’s gender	−0.31	−1.31		0.02
Child’s age	−0.18 *	−2.40		0.08
2		3	−0.19	0.02
Child’s gender	−0.17	−0.69		0.01
Child’s age	−0.14 *	−1.75		0.04
Behavioral disorder	−0.40	−1.57		0.03
3		13.02	10.03	0.32
Child’s gender	−0.10	−0.51		<0.01
Child’s age	−0.14 *	−2.14		0.04
Behavioral disorder	−0.09	−0.42		0.03
Parenting alliance	0.81 **	6.19		0.32
4		13	–0.02	0.06
Child’s gender	−0.12	−0.65		<0.01
Child’s age	−0.16 *	−2.59		0.05
Behavioral disorder	0.20	0.10		<0.01
Parenting alliance	0.49 *	2.88		0.06
Behavioral disorder X Parenting alliance	0.71 *	2.79		0.06

*sr*^2^ = predictors; Δ*R*^2^ = model; * p < 0.05, ** *p* < 0.01.

**Table 2:** Hierarchical regression model coefficients for emotional disorders and parenting alliance predicting family cohesion.

Step	β	*t/F*	Δ*F*	*^a^sr*^2^/Δ*R*^2^
1		3.19	3.19	0.06
Child’s gender	−0.31	−1.31		0.02
Child’s age	−0.18 *	−2.40		0.08
2		2.10	−1.08	−0.01
Child’s gender	−0.30	−1.30		0.02
Child’s age	−0.19 *	−2.38		0.08
Emotional disorder	−0.04	−0.15		<0.01
3		13.22	11.12	0.36
Child’ gender	−0.13	−0.69		<0.01
Child’s age	−0.14 *	−2.26		0.04
Emotional disorder	0.16	0.79		0.01
Parenting alliance	0.84 **	6.54		0.35
4		12.30	−0.92	0.04
Child’s gender	−0.02	−0.11		<0.01
Child’s age	−0.12	−1.96		<0.01
Emotional disorder	0.05	0.23		<0.01
Parenting alliance	0.99 **	7.04		0.39
Emotional disorder X Parenting alliance	−0.76 *	−2.30		0.04

*sr*^2^ = predictors; Δ*R*^2^ = model. * p < 0.05. ** *p* < 0.01.

## Data Availability

Data sharing not available due to ethical issues. Consent forms signed by participants did not include data sharing.
